# Dynamic Characteristics and Effective Stiffness Properties of Sandwich Panels with Hierarchical Hexagonal Honeycomb

**DOI:** 10.3390/ma16175741

**Published:** 2023-08-22

**Authors:** Zixuan Bai, Cheng Chen, Xinlong Yang, Yifeng Zhong, Rong Liu

**Affiliations:** 1 School of Civil Engineering, Chongqing University, Chongqing 400045, China; 20204904@cqu.edu.cn (Z.B.); chencheng@cqu.edu.cn (C.C.); 20195013@cqu.edu.cn (X.Y.); 202016131332@cqu.edu.cn (R.L.); 2 Key Laboratory of New Technology for Construction of Cities in Mountain Area, Chongqing University, Chongqing 400045, China

**Keywords:** sandwich panel, hierarchical hexagonal honeycomb, equivalent stiffness properties, dynamic characteristics, variational asymptotic analysis

## Abstract

The dynamic characteristics of sandwich panels with a hierarchical hexagonal honeycomb (SP-HHHs) show significant improvements due to their distinct hierarchy configurations. However, this also increases the complexity of structural analysis. To address this issue, the variational asymptotic method was utilized to homogenize the unit cell of the SP-HHH and obtain the equivalent stiffness, establishing a two-dimensional equivalent plate model (2D-EPM). The accuracy and effectiveness of the 2D-EPM were then verified through comparisons with the results from a detailed 3D FE model in terms of the free vibration and frequency- and time-domain forced vibration, as well as through local field recovery analysis at peak and trough times. Furthermore, the tailorability of the typical unit cell was utilized to perform a parametric analysis of the effects of the length and thickness ratios of the first-order hierarchy on the dynamic characteristics of the SP-HHH under periodic loading. The results reveal that the vertices serve as weak points in the SP-HHH, while the vertex cell pattern significantly influences the specific stiffness and stiffness characteristics of the panel. The SP-HHH with hexagonal vertex cells has superior specific stiffness compared to panels with circular and rectangular vertex cells, resulting in a more lightweight design and enhanced stiffness.

## 1. Introduction

The honeycomb sandwich structure, with its impressive mechanical properties, including high strength, low weight, and superior energy absorption capacity, has been widely employed in a range of fields, including aerospace, ships [1], and vehicles [2,3]. Due to the challenging application environments, sandwich panels’ performance requirements are demanding, which has led to extensive studies aimed at optimizing the honeycomb sandwich structures [4,5]. Significantly, both the facesheet and core layer in a sandwich panel play equally critical roles; however, it is the core layer that primarily governs the mechanical properties of the panel. Hence, researchers’ efforts to improve the mechanical properties have focused on structural changes in the core layer [6]. For instance, Wang et al. [7] suggested using a triangular honeycomb sandwich structure to simplify the construction and enhance the connection between local areas, thus improving bending resistance. Similarly, Yang et al. [8] explored the use of a core layer with a negative Poisson’s ratio to enhance the sandwich panel’s shear resistance and hardness, resulting in ductile behavior.

**Figure 1:**
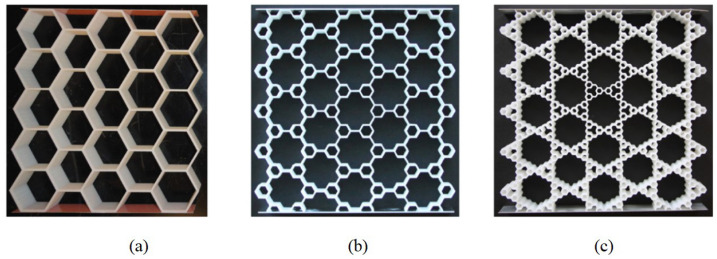
Hierarchical hexagonal honeycombs with (**a**) zero-order hierarchy, (**b**) first-order hierarchy, and (**c**) second-order hierarchy [11].

The hierarchical honeycomb sandwich structure has recently gained popularity as a result of its unique core-layer construction, which builds on the well-known honeycomb sandwich panel design [9]. This new structure is formed by replacing the vertices of regular honeycomb cores with smaller honeycomb-like units, which can themselves be further replaced with smaller ones, leading to a hierarchy of structures ranging from zero-order hierarchy (i.e., traditional honeycomb sandwich structures) to first-order hierarchy, second-order hierarchy, and so on, as illustrated in Figure 1. Researchers have examined and confirmed the superior mechanical properties of hierarchical honeycomb sandwich structures compared to traditional honeycomb sandwich structures [10]. For instance, Ajdari et al. [11] verified that the stiffness of first-order and second-order hierarchical honeycomb sandwich structures can be, respectively, 2 and 3.5 times greater than that of traditional honeycomb sandwich structures with the same mass. Zhang et al. [12] found that hierarchical honeycomb structures could improve crushing strength compared to traditional honeycomb structures.

Traditional honeycomb sandwich panels have found extensive applications in industries such as the aerospace, shipbuilding, and vehicle industries, where exceptional impact resistance is of paramount importance. However, hierarchical honeycomb sandwich structures, as indicated by Sun et al. [13], can increase specific energy absorption (SEA) by 81.3 and 185.7% for first- and second-order hierarchical honeycomb sandwich structures, respectively, thus offering superior resistance and greater potential for these industries. Furthermore, Li et al. [14] discovered that hierarchical honeycomb sandwich structures not only possess the aforementioned superior properties but also have improved bending resistance. Hence, hierarchical honeycomb sandwich structures demonstrate several exceptional characteristics, a few of which have been discussed, and possess tremendous potential for future applications.

Hierarchical honeycomb structures find broad applications in the aerospace, automotive, and maritime industries, despite the dearth of relevant research [15]. While studies have demonstrated that hierarchical honeycomb sandwich structures possess superior mechanical properties, the impact of vibrations commonly observed in practical applications on their operational status remains unclear [16,17]. Hence, it is imperative to investigate the dynamic characteristics of these structures. Numerous studies on the dynamic characteristics of honeycomb sandwich structures have been conducted. Li et al. [18] and Pham et al. [19] conducted an analysis of the free vibration of a honeycomb sandwich plate using hyperbolic tangent shear deformation theory and improved the higher-order element. Wang et al. [20] investigated the free vibration of composite sandwich layers through numerical simulations and experimental methods. However, few studies have been conducted on the dynamic characteristics of hierarchical honeycomb sandwich structures [21,22]. As a result, the dynamic characteristics of these structures and their suitability for application in the aforementioned industries remain uncertain, as do any unexpected vibration-related properties they may exhibit. Furthermore, due to the complexity of these structures, engineers require simpler research methods. This study aims to fill the existing knowledge gap by utilizing a simplified analysis method to investigate the dynamic characteristics of hierarchical honeycomb sandwich structures.

Recently, Cesnik and Hodges [23] proposed the variational asymptotic method (VAM) for the rigorous construction of reduced plate and shell models, achieving a good balance between efficiency and accuracy [24,25]. The analysis of the original 3D plate is approximated by using a constitutive model for a unit cell and conducting a corresponding 2D equivalent plate model (2D-EPM) analysis. All approximations are exclusively confined within the constitutive modeling, and the accuracy of these approximations is ensured to be optimal by the VAM. The unit cell is employed to fill the gap between the effective properties and the macro-structural analysis [26]. This study contributes to the development of a VAM-based 2D-EPM for sandwich panels with a hierarchical hexagonal honeycomb (SP-HHHs), which is achieved by replacing the vertices of a regular hexagonal honeycomb with smaller hexagons, as shown in Figure 1b. This method represents a practical and effective approach for analyzing these structures in engineering applications and fills the research gap concerning their dynamic characteristics.

This paper is structured as follows: Section 2 outlines the theoretical derivation process for establishing a VAM-based 2D-EPM for SP-HHHs. Section 3 compares and discusses the results for free and forced vibrations using both 3D and 2D models. Section 4 investigates the effects of the length and thickness ratios of the first-order hierarchy on the dynamic characteristics and effective stiffness properties of the SP-HHH. Section 5 compares the dynamic characteristics of hierarchical sandwich panels with different vertex patterns. Lastly, Section 6 presents the study’s concluding remarks.

## 2. Variational Asymptotic Equivalent Model for SP-HHH

**Figure 2:**
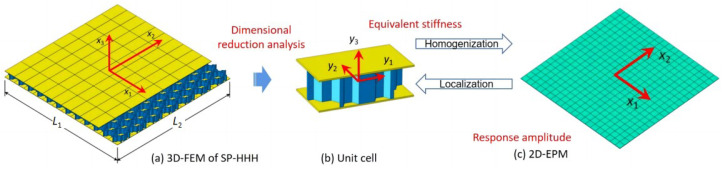
Analysis process using VAM-based equivalent model: (**a**) 3D FE model; (**b**) constitutive modeling over the unit cell; (**c**) 2D equivalent plate model.

This section details the derivation of a VAM-based 2D-EPM for the SP-HHH. The 3D-FEM of the SP-HHH is reduced to constitutive modeling over the 3D unit cell (providing effective stiffness properties) and the 2D-EPM for global analysis. Figure 2 depicts the dimension reduction analysis of the SP-HHH, where $y_{1}-y_{2}$ and $x_{1}-x_{2}$ originate from the geometric center of the core cell and the panel, respectively. It is important to note that the unit cell depicted in Figure 2b requires a division of the small hexagons located at the upper and lower vertices to ensure its periodicity.

### 2.1. Kinematics of the SP-HHH

From a macro perspective, the equivalent single layer (ESL) analysis treats a sandwich plate as a continuous medium under the assumption that displacement exhibits gradual variation across the unit cell. Accordingly, the displacement function of the SP-HHH is defined on the reference plane $x_{1}-x_{2}$ while neglecting $x_{3}$. The partial derivative of the displacement function is then determined as [27]

(1) $$\frac{\partial u\left(x_{\alpha },y_{i},t\right)}{\partial x_{\alpha }}={\left.\frac{\partial u\left(x_{\alpha },y_{i},t\right)}{\partial x_{\alpha }}\right|}_{y_{i}=const}+{\left.\frac{1}{\zeta }\frac{\partial u\left(x_{\alpha },y_{i},t\right)}{\partial y_{i}}\right|}_{x_{\alpha }=const}\equiv u_{,\alpha }+\frac{1}{\zeta }u_{;i},$$

where ${\left(\cdot \right)}_{,\alpha }$ and ${\left(\cdot \right)}_{;i}$ represent the derivatives with respect to macro-coordinates $x_{\alpha },\left(\alpha =1,2\right)$ and micro-coordinates $y_{i},\left(i=1,2,3\right)$, respectively, and ζ denotes the small parameter representing the ratio of the thickness to the in-plane dimensions.

### 2.2. Step One: 3D Strain Field

The 2D plate variables (${\bar{u}}_{i}$) and warping functions ($\chi_{i}$) can be utilized to represent the 3D displacement field ($u_{i}$) of the SP-HHH such that [28]

(2) $$\begin{array}{cc} \; & u_{1}\left(x_{\alpha },y_{i},t\right)=\underline{{\bar{u}}_{1}\left(x_{\alpha },t\right)-\zeta y_{3}{\bar{u}}_{3,1}\left(x_{\alpha },t\right)}+\chi_{1}\left(x_{\alpha },y_{i},t\right)\\ \; & u_{2}\left(x_{\alpha },y_{i},t\right)=\underline{{\bar{u}}_{2}\left(x_{\alpha },t\right)-\zeta y_{3}{\bar{u}}_{3,2}\left(x_{\alpha },t\right)}+\chi_{2}\left(x_{\alpha },y_{i},t\right)\\ \; & u_{3}\left(x_{\alpha },y_{i},t\right)=\underline{{\bar{u}}_{3}\left(x_{\alpha },t\right)}+\chi_{3}\left(x_{\alpha },y_{i},t\right) \end{array},$$

where the underlined terms denote the deformations from the classic plate theory.

From Equation (2), the explicit expressions of ${\bar{u}}_{i}$ can be obtained as

(3) $${\bar{u}}_{1}=\langle u_{1}\rangle +\langle \zeta y_{3}\rangle {\bar{u}}_{3,1},\;{\bar{u}}_{2}=\langle u_{2}\rangle +\langle \zeta y_{3}\rangle {\bar{u}}_{3,2},\;{\bar{u}}_{3}=\langle u_{3}\rangle ,$$

where 〈·〉 denotes the volume average over the 3D unit cell.

The 2D displacements can be calculated as the average of the corresponding 3D displacements using Equation (3), and the warping functions are constrained accordingly by

(4) $$\langle \chi_{i}\rangle =0.$$

The strain field in the 3D-FEM can be expressed by decomposing the rotation tensor in a suitable manner, such as

(5) $$\varepsilon_{ij}=\frac{1}{2}\left(\frac{\partial u_{i}}{\partial x_{j}}+\frac{\partial u_{j}}{\partial x_{i}}\right)-\delta_{ij},$$

where $\delta_{ij}$ is the Kronecker symbol,

The required warping functions are updated based on the variable change according to Equation (1),

(6) $$\chi_{i}\left(x_{1},x_{2},x_{3}\right)=v_{i}\left(x_{1},x_{2},x_{3}\right)+\zeta y_{3}\varphi_{i}\left(y_{1},y_{2},y_{3}\right),$$

where $v_{i}$ and $\varphi_{i}$ represent global and local warping functions in the macro- and micro-coordinate systems, respectively.

Substituting Equations (2) and (6) into Equation (5) and eliminating higher-order terms with no effect on the total energy, we can derive the explicit formulation for the 3D strain field as

(7) $$\begin{array}{cc} \; & \varepsilon_{11}=\epsilon_{11}+\zeta y_{3}\kappa_{11}+\zeta v_{1,1}+\varphi_{1;1},\\ \; & 2\varepsilon_{12}=2\epsilon_{12}+\zeta y_{3}\left(\kappa_{12}+\kappa_{21}\right)+\zeta \left(v_{2,1}+v_{1,2}\right)+\varphi_{1;2}+\varphi_{2;1},\\ \; & \varepsilon_{22}=\epsilon_{22}+\zeta y_{3}\kappa_{22}+\zeta v_{2,2}+\varphi_{2;2},\\ \; & 2\varepsilon_{13}=\zeta v_{3,1}+\varphi_{3;1}+\varphi_{1;3},\\ \; & 2\varepsilon_{23}=\zeta v_{3,2}+\varphi_{3;2}+\varphi_{2;3,},\\ \; & \varepsilon_{33}=\varphi_{3;3}, \end{array}$$

where

(8) $$\epsilon_{\alpha \beta }\left(x_{1},x_{2}\right)=\frac{1}{2}\left({\bar{u}}_{\alpha ,\beta }+{\bar{u}}_{\beta ,\alpha }\right),\;\kappa_{\alpha \beta }\left(x_{1},x_{2}\right)=-{\bar{u}}_{3,\alpha \beta }.$$

The 3D strain field E can be expressed in matrix form, such as

(9) $$\begin{array}{c} E_{e}={\left[\varepsilon_{11}\;\varepsilon_{22}\;2\varepsilon_{12}\right]}^{T}=\epsilon +\zeta y_{3}\kappa +I_{\alpha }\left(\varphi_{∥;\alpha }+\zeta v_{∥,\alpha }\right),\\ 2E_{s}={\left[2\varepsilon_{13}\;2\varepsilon_{23}\right]}^{T}=\zeta v_{∥,3}+e_{\alpha }\left(\varphi_{3;\alpha }+\varphi_{\alpha ;3}\right),\\ E_{t}=\varepsilon_{33}=\varphi_{3;3}, \end{array}$$

where ${\left(\right)}_{\left|\right|}={\left[{\left(\right)}_{1}\;{\left(\right)}_{2}\right]}^{T}$, $\epsilon ={\left[\begin{array}{ccc} \epsilon_{11} & 2\epsilon_{12} & \epsilon_{22} \end{array}\right]}^{T}$, $\kappa ={\left[\kappa_{11}\;\kappa_{12}+\kappa_{21}\;\kappa_{22}\right]}^{T}$, and

(10) $$I_{1}=\left[\begin{array}{cc} 1 & 0\\ 0 & 1\\ 0 & 0 \end{array}\right],\;I_{2}=\left[\begin{array}{cc} 0 & 0\\ 1 & 0\\ 0 & 1 \end{array}\right],\;e_{1}=\left\{\begin{array}{c} 1\\ 0 \end{array}\right\},\;e_{2}=\left\{\begin{array}{c} 0\\ 1 \end{array}\right\}.$$

### 2.3. Step Two: 3D Energy Expression

The strain energy of the SP-HHH is

(11) $$U=\underset{s}{\;\int \int \;}\frac{1}{\Omega }U_{\Omega }dx_{1}dx_{2},$$

where *s* refers to the reference surface of the sandwich panel; $\frac{U_{\Omega }}{\Omega }$ is the strain energy density, with Ω representing the projected area of the unit cell in the $y_{1}-y_{2}$ plane; and $U_{\Omega }$ is the strain energy of a unit cell, which can be expressed as the summation of the energy stored in each member of the unit cell,

(12) $$\begin{array}{cc} \; & U_{\Omega }=\int_{-\frac{t_{c}}{2}}^{-\frac{t_{c}}{2}-t_{f}}\int_{-\frac{\sqrt{3}}{2}l_{1}}^{\frac{\sqrt{3}}{2}l_{1}}\int_{-\frac{3}{2}l_{1}}^{\frac{3}{2}l_{1}}E_{b}^{T}D_{b}E_{b}dy_{1}dy_{2}dy_{3}+\int_{\frac{t_{c}}{2}}^{\frac{t_{c}}{2}+t_{f}}\int_{-\frac{\sqrt{3}}{2}l_{1}}^{\frac{\sqrt{3}}{2}l_{1}}\int_{-\frac{3}{2}l_{1}}^{\frac{3}{2}l_{1}}E_{t}^{T}D_{t}E_{t}dy_{1}dy_{2}dy_{3}\\ \; & +6\times \int_{-\frac{t_{c}}{2}}^{\frac{t_{c}}{2}}\int_{-\frac{t_{1}}{2}}^{\frac{t_{1}}{2}}\int_{-\frac{l_{1}-2l_{2}}{2}}^{\frac{l_{1}-2l_{2}}{2}}E_{A}^{T}D_{A}E_{A}dy_{1}^{\prime }dy_{2}^{\prime }dy_{3}^{\prime }+24\times \int_{-\frac{t_{c}}{2}}^{\frac{t_{c}}{2}}\int_{-\frac{t_{2}}{2}}^{\frac{t_{2}}{2}}\int_{-\frac{l_{2}}{2}}^{\frac{l_{2}}{2}}E_{B}^{T}D_{B}E_{B}dy_{1}^{\prime }dy_{2}^{\prime }dy_{3}^{\prime }\\ \; & +2\times \int_{-\frac{l_{c}}{2}}^{\frac{t_{c}}{2}}\int_{-\frac{t_{1}}{2}}^{\frac{t_{1}}{2}}\int_{-\frac{l_{1}-2l_{2}}{4}}^{\frac{l_{1}-2l_{2}}{4}}E_{C}^{T}D_{C}E_{C}dy_{1}^{\prime }dy_{2}^{\prime }dy_{3}^{\prime } \end{array},$$

**Figure 3:**
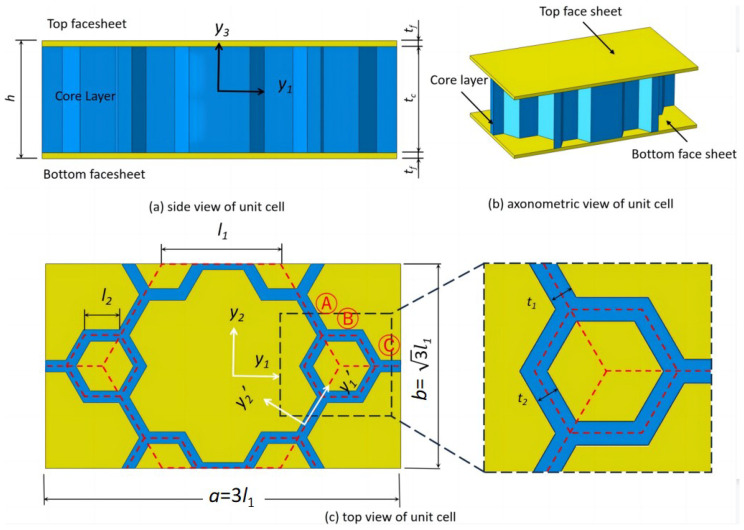
Unit cell of the SP-HHH and its parts for the strain energy integral ($l_{1}$ and $l_{2}$ are side lengths of the microstructure and hexagon, respectively; $t_{1}$ and $t_{2}$ correspond to their respective wall thicknesses; $t_{f}$ and $t_{c}$ are the thicknesses of the facesheet and core layer, respectively).

Here, the subscripts *b* and *t* represent the bottom and top facesheets of the sandwich panel, respectively; the subscripts *A*, *B*, and *C* denote the corresponding members of the core cell, as shown in Figure 3c; and $y_{i}^{\prime }$ represents the local element coordinates, and its origin and orientation vary with changes in each cell wall.

Equation (11) can be written as

(13) $$U=\frac{1}{2}\langle E^{T}DE\rangle =\frac{1}{2}\langle {\left\{\begin{array}{c} E_{e}\\ 2E_{s}\\ E_{t} \end{array}\right\}}^{T}\left[\begin{array}{ccc} D_{e} & D_{es} & D_{et}\\ D_{es}^{T} & D_{s} & D_{st}\\ D_{et}^{T} & D_{st}^{T} & D_{t} \end{array}\right]\left\{\begin{array}{c} E_{e}\\ 2E_{s}\\ E_{t} \end{array}\right\}\rangle ,$$

where $D_{e},D_{es},D_{et},D_{s},D_{st}$, and $D_{t}$ are the sub-matrices of the 6×6 material matrix.

The virtual work done by the external load is

(14) $$\begin{array}{c} W_{3D}=W_{2D}+W^{*}\\ \;=\int_{s}\left(p_{i}{\bar{u}}_{i}+q_{\alpha }\delta {\bar{u}}_{3,\alpha }\right)ds+\int_{s}\left(\langle f_{i}v_{i}\rangle +\tau_{i}h\varphi_{i}/2-\beta_{i}h\varphi_{i}/2\right)ds, \end{array}$$

where $\beta_{i}$ and $\tau_{i}$ are the traction forces on the bottom and top surfaces, respectively; $f_{i}$ denotes the body force; and $p_{i}=\langle f_{i}\rangle +\tau_{i}+\beta_{i}$, $q_{\alpha }=h/2\left(\beta_{\alpha }-\tau_{\alpha }\right)-\langle \zeta y_{3}f_{\alpha }\rangle$.

The 3D kinetic energy can be decomposed into the kinetic energy of the 2D-EPM and the residual kinetic energy that cannot captured by the 2D-EPM, such as

(15) $$K_{3D}=K_{2D}+K^{*},$$

where

(16) $$K_{2D}=\frac{1}{2}\int_{s}\left(\rho^{*}V^{T}V+2\omega^{T}\rho^{*}\xi V+\omega^{T}\Phi \omega \right)ds,$$

(17) $$K^{*}=\frac{1}{2}\int_{V}\rho \left[{\left(\omega \chi +\dot{\chi }\right)}^{T}\left(\omega \chi +\dot{\chi }\right)+2{\left(V+\omega \xi \right)}^{T}\left(\omega \chi +\dot{\chi }\right)\right]dV,$$

where *V* represents the absolute velocity measured on the deformed reference surface; ω denotes the inertial angular velocity; V represents the volume domain enclosed by the panel’s boundaries or surfaces; $\dot{\chi }=\partial \chi /\partial t$, $\xi ={\left[\begin{array}{ccc} 0 & 0 & x_{3} \end{array}\right]}^{T}$; $\rho^{*}\xi ={\left[\begin{array}{ccc} 0 & 0 & \langle x_{3}\rho \rangle \end{array}\right]}^{T}$; $\Phi =\left[\begin{array}{ccc} \langle x_{3}^{2}\rho \rangle & 0 & 0\\ 0 & \langle x_{3}^{2}\rho \rangle & 0\\ 0 & 0 & 0 \end{array}\right]$; ρ and $\rho^{*}$ are the density and equivalent density of the 3D-FEM and 2D-EPM, respectively; and $\rho^{*}$ can be determined as in step five.

The elastodynamic behavior of a sandwich panel is governed by the principle of minimum potential energy, which can be written as follows

(18) $$\int_{t_{1}}^{t_{2}}\left[\delta \left(K_{2D}+K^{*}-U\right)+{\bar{\delta W}}_{2D}+{\bar{\delta W}}^{*}\right]dt=0,$$

and the variational principle governing $\chi_{i}$ can be written as the principle of the stationary potential energy,

(19) $$\min_{\chi_{i}\in \mathrm{Equation}\;\left(4\right)}\langle E^{T}DE\rangle .$$

It is evident that this variational problem for $\chi_{i}$ is presented exclusively over the unit cell rather than the entire structure. The solution for $\chi_{i}$ can be derived by asymptotic analysis of the variational statement in Equation (18) without relying on ad hoc assumptions. The asymptotic analysis involves expanding the displacement field and other relevant variables in a series of small parameters or perturbation parameters associated with the characteristic length scales or geometrical features of the unit cell.

### 2.4. Step Three: Asymptotic Analysis

Before applying the VAM, it is necessary to evaluate the order of each term in Equation (18)

(20) $$\begin{array}{cc} \; & \epsilon_{\alpha \beta }∼h\kappa_{\alpha \beta }∼\varphi_{i}∼n,v_{i}∼hn,v_{i,\alpha }∼\frac{h}{a}n,v_{,3}∼n\\ \; & hf_{\alpha }∼\alpha_{\alpha }∼\beta_{\alpha }∼\mu \frac{h}{a}n,hf_{3}∼\alpha_{3}∼\beta_{3}∼\mu {\left(\frac{h}{a}\right)}^{2}, \end{array}$$

where μ denotes the order of material properties, which signifies the significance of the material property for the overall behavior; *n* represents the order of the minimum strain, which specifies the power to which the minimum strain is raised in the analysis.

To obtain the zeroth-order approximation of the variational statement in Equation (18), the asymptotically smaller terms (namely, $K^{*}$ and ${\bar{\delta W}}^{*}$) are eliminated,

(21) $$\int_{t_{1}}^{t_{2}}\left[\delta \left(K_{2D}-\int_{\Omega }U_{0}d\Omega \right)+{\bar{\delta W}}_{2D}\right]dt=0,$$

where $U_{0}$ can be obtained from Equation (13) by removing the derivatives of the displacement field with respect to $x_{\alpha }$,

(22) $$2U_{0}==\langle \begin{array}{c} {\left(\epsilon +\zeta y_{3}\kappa \right)}^{T}D_{e}\left(\epsilon +\zeta y_{3}\kappa \right)+2{\left(\epsilon +\zeta y_{3}\kappa \right)}^{T}\left(D_{es}\zeta v_{∥,3}+D_{et}\varphi_{3;3}\right)\\ +\zeta v_{∥,3}^{T}D_{s}\zeta v_{∥,3}+2\zeta v_{∥,3}^{T}D_{st}\varphi_{3;3}+\varphi_{3;3}^{T}D_{t}\varphi_{3;3} \end{array}\rangle .$$

The introduction of Lagrange multipliers $\lambda_{i}$ allows us to derive the corresponding Euler–Lagrange equation as

(23) $$\begin{array}{cc} \; & {\left[{\left(\epsilon +\zeta y_{3}\kappa \right)}^{T}D_{es}+\zeta v_{∥,3}^{T}D_{s}+\zeta v_{∥,3}^{T}D_{st}\right]}_{,3}=\lambda_{∥},\\ \; & {\left[{\left(\epsilon +\zeta y_{3}\kappa \right)}^{T}D_{et}+\zeta v_{∥,3}^{T}D_{st}+\varphi_{3;3}D_{t}\right]}_{,3}=\lambda_{3}, \end{array}$$

where $\lambda_{\left|\right|}={\left[\lambda_{1}\;\lambda_{2}\right]}^{T}$.

The stress-free boundary conditions at the top and bottom surfaces can be obtained as

(24) $$\begin{array}{cc} \; & {\left[{\left(\epsilon +\zeta y_{3}\kappa \right)}^{T}D_{es}+\zeta v_{∥,3}^{T}D_{s}+\zeta v_{∥,3}^{T}D_{st}\right]}^{+/-}=0,\\ \; & {\left[{\left(\epsilon +\zeta y_{3}\kappa \right)}^{T}D_{et}+\zeta v_{∥,3}^{T}D_{st}+\varphi_{3;3}D_{t}\right]}^{+/-}=0. \end{array}$$

From these conditions, the solutions for $v_{\left|\right|}$ and $v_{3}$ can be obtained as

(25) $$v_{∥}={\langle -\left(\epsilon +\zeta y_{3}\kappa \right){\bar{D}}_{es}{\left(D_{s}\right)}^{-1}\rangle }^{T},v_{3}=\langle -\left(\epsilon +\zeta y_{3}\kappa \right){\bar{D}}_{et}{\left({\bar{D}}_{t}\right)}^{-1}\rangle ,$$

where

(26) $$\begin{array}{c} {\bar{D}}_{es}=D_{es}-{\bar{D}}_{et}{\left(D_{st}\right)}^{T}{\left({\bar{D}}_{t}\right)}^{-1},\;{\bar{D}}_{et}=D_{et}-D_{es}{\left(D_{s}\right)}^{-1}D_{st},\\ {\bar{D}}_{t}=D_{t}-{\left(D_{st}\right)}^{T}{\left(D_{s}\right)}^{-1}D_{st}. \end{array}$$

Substituting Equation (25) into Equation (22), the stain energy of the 2D-EPM can be obtained as

(27) $$\begin{array}{c} U_{2D}=\frac{1}{2}\langle {\left(\epsilon +\zeta y_{3}\kappa \right)}^{T}K\left(\epsilon +\zeta y_{3}\kappa \right)\rangle =\frac{1}{2}{\left\{\begin{array}{c} \epsilon \\ \kappa \end{array}\right\}}^{T}\left[\begin{array}{cc} A & B\\ B^{T} & D \end{array}\right]\left\{\begin{array}{c} \epsilon \\ \kappa \end{array}\right\} \end{array},$$

where

(28) $$\begin{array}{c} A=\langle K\rangle ,B=\langle \zeta y_{3}K\rangle ,D=\langle \zeta y_{3}^{2}K\rangle ,\\ K=D_{e}-{\bar{D}}_{es}D_{s}^{-1}D_{es}^{T}-{\bar{D}}_{et}D_{et}^{T}/{\bar{D}}_{t}. \end{array}$$

The constitutive equation of the 2D-EPM can be expressed as

(29) $$\left\{\begin{array}{c} N_{11}\\ N_{22}\\ N_{12}\\ M_{11}\\ M_{22}\\ M_{12} \end{array}\right\}=\left[\begin{array}{cccccc} A_{11} & A_{12} & A_{16} & B_{11} & B_{12} & B_{16}\\ A_{12} & A_{22} & A_{26} & B_{12} & B_{22} & B_{26}\\ A_{16} & A_{26} & A_{66} & B_{16} & B_{26} & B_{66}\\ B_{11} & B_{12} & B_{16} & D_{11} & D_{12} & D_{16}\\ B_{12} & B_{22} & B_{26} & D_{12} & D_{22} & D_{26}\\ B_{16} & B_{26} & B_{66} & D_{16} & D_{26} & D_{66} \end{array}\right]\left\{\begin{array}{c} \epsilon_{11}^{*}\\ \epsilon_{22}^{*}\\ 2\epsilon_{12}^{*}\\ \kappa_{11}^{*}\\ \kappa_{22}^{*}\\ 2\kappa_{12}^{*} \end{array}\right\}.$$

where $A_{11}$ and $A_{22}$, respectively, indicate the tensile stiffnesses along the $x_{1}$ and $x_{2}$ directions, $A_{66}$ indicates the in-plane shear stiffness, $A_{12}$ and $A_{21}$ indicate the tensile couplings, and $A_{16}$ and $A_{61}$, as well as $A_{26}$ and $A_{62}$, indicate the tensile-shear couplings; $D_{11}$ and $D_{22}$, respectively, indicate the bending stiffnesses along the $x_{1}$ and $x_{2}$ directions, $D_{66}$ indicates the torsional stiffness, $D_{12}$ and $D_{21}$ indicate the bending couplings, and $D_{26}$ and $D_{62}$, as well as $D_{16}$ and $D_{61}$, indicate the bending-torsional couplings; and $B_{11}$ and $B_{22}$ indicate the tensile-bending couplings, $B_{16}$ indicates the tensile-torsional coupling, $B_{26}$ indicates the bending-shear coupling, and $B_{66}$ indicates the torsional-shear coupling.

The obtained constitutive relationship can be incorporated into the two-dimensional equivalent plate to facilitate free vibration analysis, allowing for the determination of natural frequencies and modes. Additionally, with the acquired damping system, forced vibration analysis can be conducted in both the time and frequency domains. This can be achieved by utilizing modal/steady-state dynamics solvers in finite element software, such as ABAQUS/Standard.

### 2.5. Step Four: Recovery Relationship

To enhance the developed model, it is imperative to determine the recovery relationship for local fields after obtaining the response amplitude. Since every material point of the 2D-EPM has an associated unit cell as its microstructure, Equation (3) can be employed for recovering the local displacement field within the unit cell,

(30) $$u_{i}={\bar{u}}_{i}+\left[\begin{array}{ccc} {\bar{u}}_{1,1} & {\bar{u}}_{1,2} & {\bar{u}}_{1,3}\\ {\bar{u}}_{2,1} & {\bar{u}}_{2,2} & {\bar{u}}_{2,3}\\ {\bar{u}}_{3,1} & {\bar{u}}_{3,2} & {\bar{u}}_{3,3} \end{array}\right]\left\{\begin{array}{c} y_{1}\\ y_{2}\\ y_{3} \end{array}\right\}+\zeta \chi_{i}.$$

The local strain field can be recovered as follows

(31) $$E_{e}^{0}={\left[\begin{array}{ccc} \varepsilon_{11}^{0} & \varepsilon_{22}^{0} & 2\varepsilon_{12}^{0} \end{array}\right]}^{T}=\epsilon +\zeta y_{3}\kappa ,\;2E_{s}^{0}={\left[\begin{array}{cc} 2\varepsilon_{13}^{0} & 2\varepsilon_{23}^{0} \end{array}\right]}^{T}=-\chi_{∥,3},\;E_{t}^{0}=\varepsilon_{33}^{0}=\chi_{3,3}.$$

The local stress field can be determined by applying the general Hooke’s law,

(32) σ=KE.

### 2.6. Step Five: Equivalent Density of 2D-EPM

The facesheets and core layer are considered to have perfect bonding without any relative sliding, allowing for the computation of the total mass of the unit cell within the SP-HHH as

(33) $$\begin{array}{cc} \; & m=m_{c}+m_{f}\\ \; & =\rho_{c}\times \left[6\times \left(l_{1}-2l_{2}\right)\times t_{1}+24\times l_{2}\times t_{2}+\left(l_{1}-2l_{2}\right)\times t_{1}\right]\times t_{c}+2\rho_{f}\times 3\sqrt{3}l_{1}^{2}\times t_{f}, \end{array}$$

where $\rho_{f}$ represents the density of the facesheet, $\rho_{c}$ denotes the density of the core layer, and $t_{f}$ and $t_{c}$ correspond to their respective thicknesses.

The equivalent density of the SP-HHH can be obtained as

(34) $$\rho^{*}=\frac{m}{V_{\Omega }}=\frac{m}{3\sqrt{3}l_{1}^{2}\times \left(t_{c}+2t_{f}\right)}.$$

## 3. Model Verification

**Figure 4:**
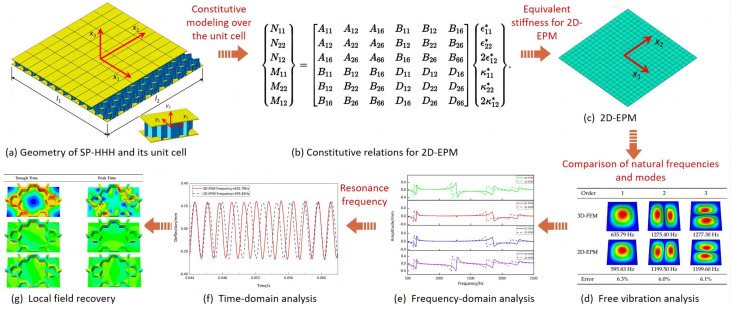
Flow chart for verifying the dynamic characteristics of the SP-HHH using the 2D-EPM.

The flowchart illustrating the validation process for the dynamic analysis using the 2D-EPM is presented in Figure 4. The equivalent stiffness properties of the SP-HHH were determined through VAM-based homogenization of the unit cell. These properties were then input into the 2D-EPM for conducting free vibration analysis. Based on the obtained natural frequencies and modes, the frequency scanning range, as well as the positions for excitation and reception in the harmonic response analysis, was determined. Subsequently, by examining the maximum resonant responses at peak and trough times, local displacement and stress fields within the unit cell were obtained using recovery relationships. To evaluate the accuracy of the 2D-EPM, the natural frequencies, modes, frequency response function (FRF) curves, and displacement–time curves at the first resonance frequency were compared with those obtained from the 3D-FEM. This comparative analysis served as the foundation for predicting the fatigue life of the SP-HHH when subjected to periodic loads.

The three-dimensional model of the SP-HHH comprised 24 unit cells in the $x_{1}$ direction and 14 unit cells in the $x_{2}$ direction. The sandwich panel had uniform side lengths of 400 mm, and the geometric parameters of the unit cell were a=30mm, $b=10\sqrt{3}\;\mathrm{mm},\;$$l_{1}=10\;\mathrm{mm},\;$$l_{2}=2\;\mathrm{mm},\;t_{1}=t_{2}=1\;\mathrm{mm},\;t_{c}=9\;\mathrm{mm}$, and $t_{f}=1\;\mathrm{mm}$. The material used in the simulation was aluminum, with material parameters of $\rho =2.7\times 10^{3}\;\mathrm{kg}/m^{3},\;$E=70GPa, and v=0.3.

**Figure 5:**
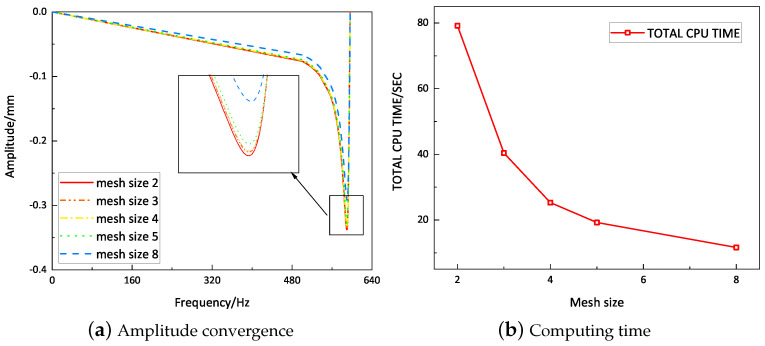
Convergence analysis of mesh size using the vibration amplitude.

**Figure 6:**
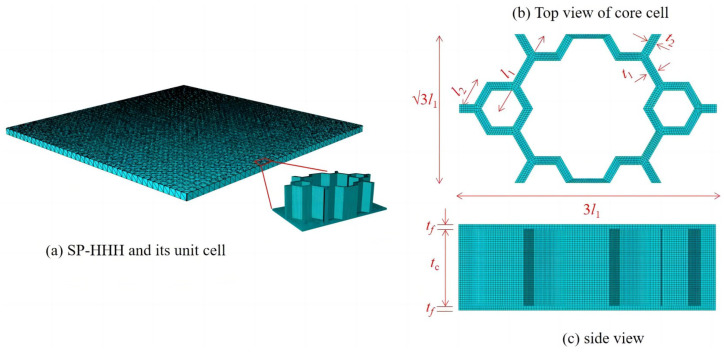
Geometry and meshing of the SP-HHH and its unit cell.

To evaluate the precision of mesh dividing, the amplitude convergence criterion was used for mesh convergence analysis. The amplitude convergence results for the model with different mesh sizes are presented in Figure 5. The model with a mesh size of eight exhibited insufficient accuracy. For mesh sizes of two, three, four, and five, the model’s calculation results were relatively close, but the computation time for mesh sizes of two and three was significantly longer compared to the mesh size of five. Therefore, to enhance computational efficiency, this study opted for a mesh size of five. The 2D-EPM employed 21,656 S4R shell elements, while the 3D-FEM utilized 255,104 C3D10 solid elements, as shown in Figure 6, both of which satisfy the accuracy requirements for engineering purposes. To construct a feasible FE model, all internal boundaries were eliminated. Furthermore, both the core layer and facesheet were assigned a solid homogeneous section with elastic properties.

### 3.1. Free Vibration Response

**Figure 7:**
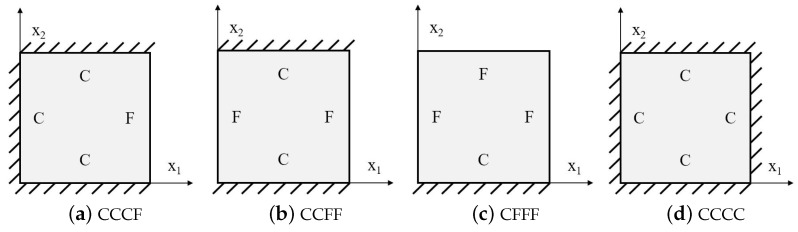
Boundary conditions adopted in the free vibration analysis.

To assess the accuracy of the 2D-EPM in predicting the natural frequencies and modes of the SP-HHH, free vibration analyses were performed with both models using various boundary conditions (BCs), as illustrated in Figure 7. The BCs included a free boundary (denoted as F) and clamped boundary (denoted as C). The deviation between the results obtained from the 3D-FEM and 2D-EPM was quantified as the “Error”, which was calculated as

(35) $$\;\mathrm{Error}\;=\left|\frac{\omega^{2D\text{-}\mathrm{EPM}}-\omega^{3D\text{-}\mathrm{FEM}}}{\omega^{3D\text{-}\mathrm{FEM}}}\right|\times 100\%$$

where $\omega^{3D-\mathrm{FEM}}$ and $\omega^{2D-\mathrm{EPM}}$ represent the natural frequencies obtained from the 3D-FEM and 2D-EPM, respectively.

Table 1 compares the first six natural frequencies of the SP-HHH as predicted by both the 3D-FEM and 2D-EPM, considering the BCs CCCF, CCFF, and CFFF. The obtained results suggested a general agreement between the natural frequencies predicted by the two models. However, an increase in the relative error of the 2D-EPM’s natural frequency predictions was observed as the order of the frequency increased. The maximum errors for the three considered BCs were 5.38%, 6.58%, and 5.32%, respectively, all of which remained below 7%. Comparative analysis of the natural frequencies obtained from the 2D-EPM and 3D-FEM, utilizing various BCs, revealed an upward trend in the natural frequencies for both models with enhanced boundary constraints.

Table 2 compares the first six natural frequencies and corresponding modes of the SP-HHH as predicted by both the 3D-FEM and 2D-EPM under CCCC BCs. The findings revealed a strong level of agreement between the natural frequencies obtained from the 2D-EPM and the 3D-FEM, with a discrepancy of only 6.3%. This value falls well within the acceptable engineering tolerance, highlighting the reliability and accuracy of the predictions. The mode complexity increased with the ascending mode order, as did the half-wavelengths in both the $x_{1}$ and $x_{2}$ directions. The modes predicted by both the 3D-FEM and 2D-EPM exhibited remarkable similarity. Specifically, the first four modes, denoted f(m,n), under the CCCC BCs corresponded to (1, 1), (2, 1), (1, 2), and (2, 2). Here, *m* and *n* represent the half-wavelengths along the $x_{1}$ and $x_{2}$ directions, respectively.

In terms of modeling efficiency, the 2D-EPM demonstrated superior performance compared to the 3D-FEM, which was primarily attributed to its simplified implementation of boundary conditions and loads. Additionally, the 2D-EPM proved to be more efficient in conducting free vibration analysis, with a computation time of 45 s when employing eight CPUs. The computing platform utilized for these analyses was a Precision Tower 7910 (Dell company, Round Rock, TX, USA) equipped with two Intel Xeon E5-2660 CPUs operating at a clock speed of 3.2 GHz with 128 GB of memory. In summary, the 2D-EPM was capable of predicting the natural frequencies and modes of the SP-HHH and was computationally superior to the 3D-FEM, which highlights its significance in the preliminary design of SP-HHHs.

### 3.2. Frequency-Domain Analysis of Forced Vibration

**Figure 8:**
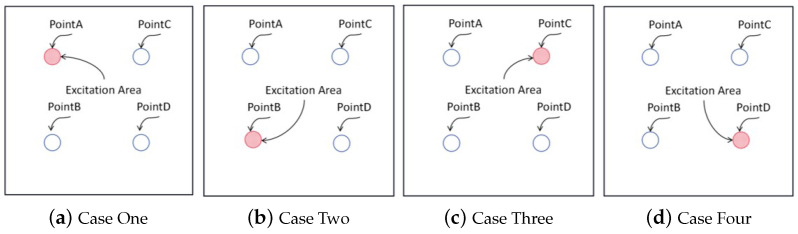
Cases used in frequency-domain analysis of forced vibration. The coordinates of points A, B, C, and D are (−100, 100), (−100, −100), (100, 100), and (100, −100), respectively.

Based on the results of the free vibration analysis, the first six natural frequencies of the sandwich panel were obtained. To validate the accuracy of the 2D-EPM in forced vibration analysis, the frequency response was investigated by conducting frequency-domain analysis. To obtain comprehensive data, four regions were designated on the sandwich panel depicted in Figure 8 for excitation and measurement of vibration amplitude. This resulted in four distinct cases with each case having a region assigned as the excitation zone, while vibration measurements were obtained from all four regions to elucidate structural responses. To prevent stress concentration, a uniform load with an amplitude of 1 MPa was distributed over each region. Based on the free vibration outcomes, frequency-domain analysis was employed in the frequency range between 500 and 3000 Hz for a 400 mm by 400 mm plate subject to CCCC BCs.

**Figure 9:**
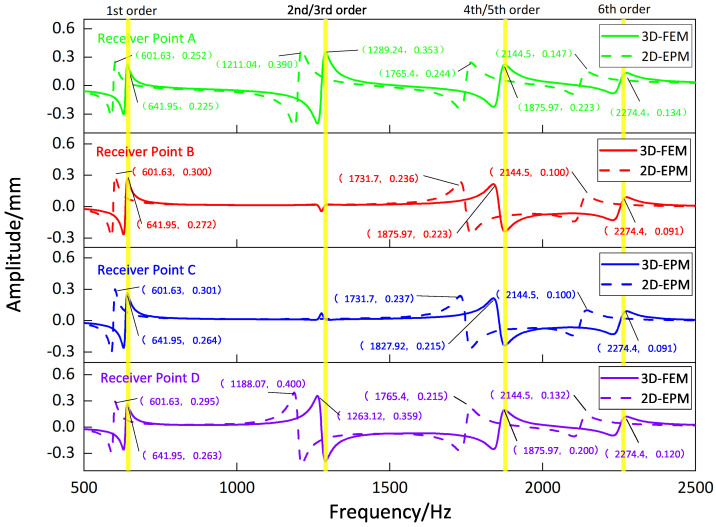
Comparison of frequency response function (FRF) curves predicted by two models for each receiver point (case one).

**Figure 10:**
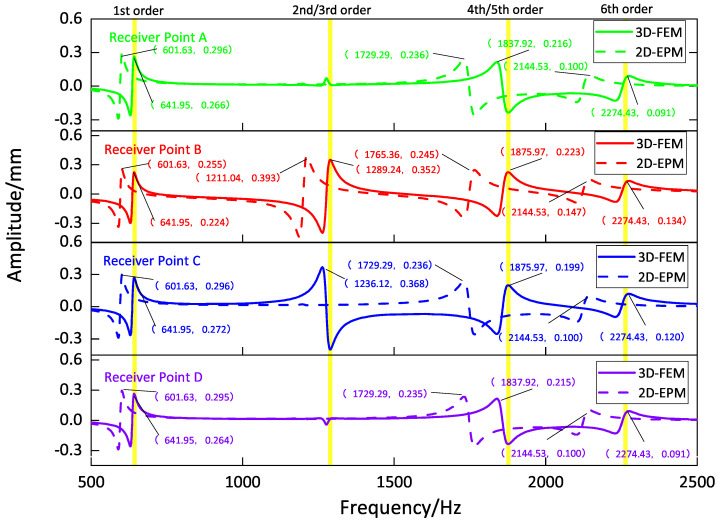
Comparison of frequency response function (FRF) curves predicted by two models for each receiver point (case two).

**Figure 11:**
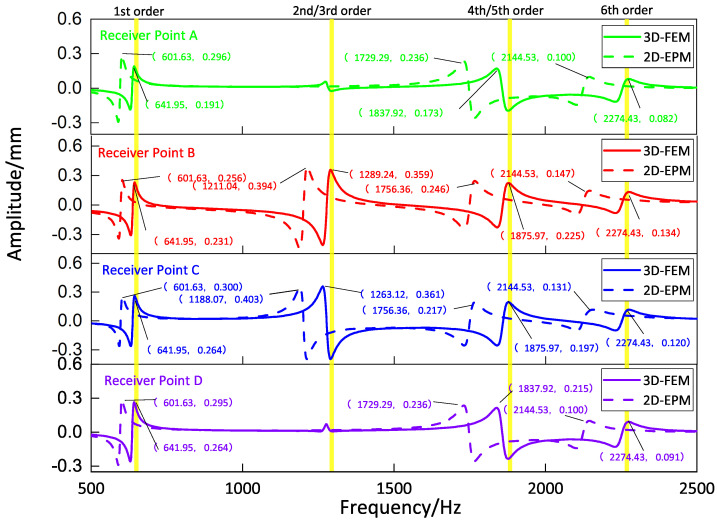
Comparison of frequency response function (FRF) curves predicted by two models for each receiver point (case three).

**Figure 12:**
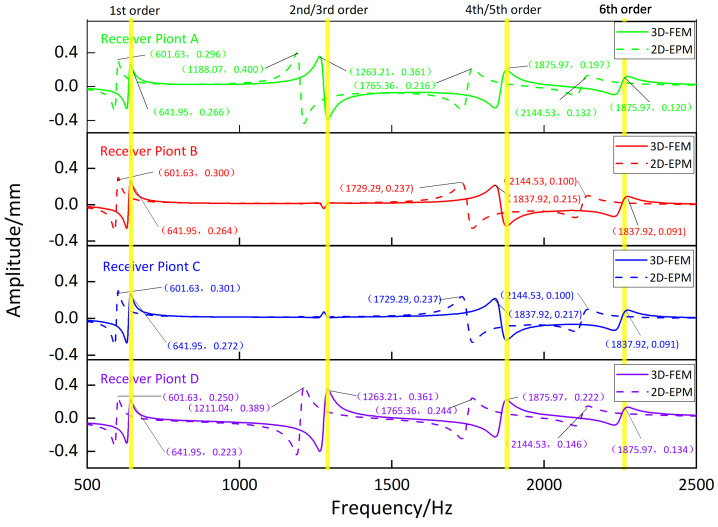
Comparison of frequency response function (FRF) curves predicted by two models for each receiver point (case four).

Figure 9, Figure 10, Figure 11 and Figure 12 compare the forced vibration responses in cases one to four predicted by the 3D-FEM and 2D-EPM, respectively. Frequency-domain analysis revealed distinctive peaks spanning from the fundamental frequency of 649 Hz to the sixth natural frequency of 2274 Hz, indicating a resonance phenomenon. Notably, the resonance peak associated with the first to the fourth natural frequencies was considerably larger than that corresponding to the fifth and sixth natural frequencies. The characteristics exhibited by the FRF curves were in good agreement with the aforementioned modal diagram under CCCC BCs. Firstly, the symmetry of the vibration response at each point corresponded to the symmetry observed in the modal diagram. For instance, in the modal diagram of the first mode, the receiving points exhibited identical displacements, leading to a high level of coherence in the FRF curve across these four points (with slightly larger amplitudes of excitation points). Similarly, the points situated on the diagonal of the fourth modal diagram displayed consistent displacement responses, causing convergence of the vibration responses of points A and D, as well as B and C, on the FRF curve. This pattern of symmetry extended to other modes as well. Secondly, owing to the close similarity between the second and third natural frequencies, the FRF curves displayed their superposition. The modes’ symmetry further contributed to this phenomenon, as points located on one diagonal effectively canceled each other out, resulting in minimal peaks, while points on the opposite diagonal overlapped, leading to larger peaks.

### 3.3. Time-Domain Analysis of Forced Vibration

The frequency-domain analysis in the previous section revealed that the central point of the panel exhibited the strongest resonance response when excited at point A in case one. To delve deeper into the resonance response in case one, we performed steady-state time-domain analyses employing both the 3D-FEM and 2D-EPM. The excitation load and modal damping ratio were selected to align with the values obtained from the frequency-domain analysis, specifically corresponding to a frequency of 635.79 Hz for the 3D-FEM and 595.83 Hz for the 2D-EPM.

**Figure 13:**
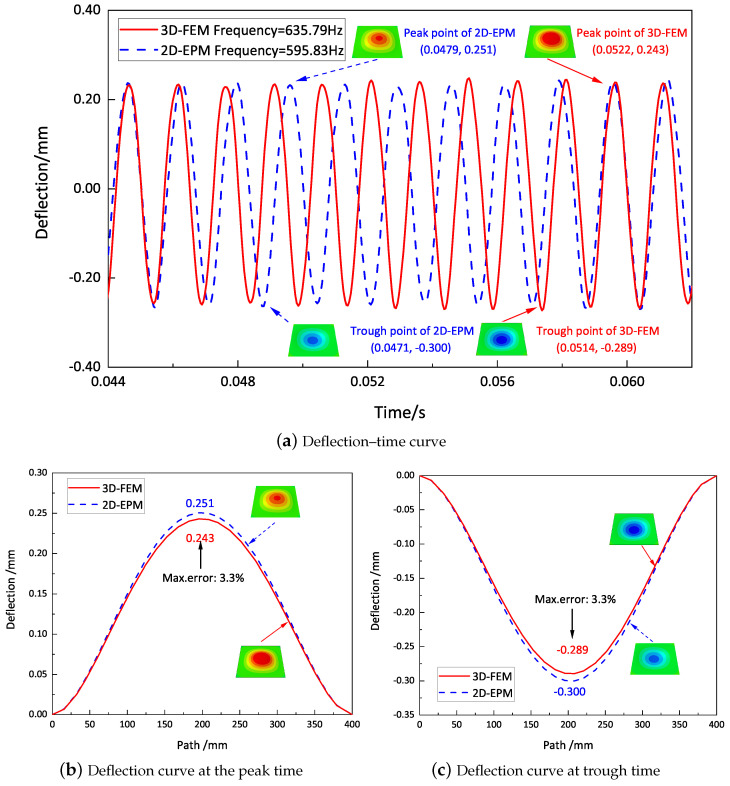
Deflection–time curves at first resonance frequency of the forced vibration response predicted by the 3D-FEM and 2D-EPM.

Figure 13a illustrates the deflection–time curves at the first resonance frequency of the forced vibration response as predicted by the 3D-FEM and 2D-EPM. The maximum displacements recorded for the 3D-FEM and 2D-EPM were 0.251 mm and 0.243 mm, respectively, with a relative error of 3.3%, which satisfies engineering accuracy requirements. Furthermore, the distinct periods observed between the 3D-FEM and 2D-EPM implied that variations in dimensions yield distinct numbers and characteristics for the vibration modes. The 3D-FEM accounted for the through-the-thickness deformation and the non-uniformity of complex structures, leading to a higher first resonance frequency compared to the 2D-EPM. The deflections measured at the peak and trough times, extracted from the deflection–time curves, are represented in Figure 13b,c, respectively. It is clear that these deflections exhibit similarities with the first free vibration mode analyzed in Table 2, thereby demonstrating a remarkable agreement between the deflection results obtained by the 3D-FEM and 2D-EPM.

### 3.4. Local Field Recovery

In this section, the displacement and strain information obtained from the point with the greatest amplitude in the 2D-EPM is used to recover the local displacement and strain field within the 3D unit cell. The recovery point corresponds to excitation point A identified in the previous time-domain analysis.

Table 3 presents the recovered displacement fields within the unit cell at the peak and trough times of the time-domain curve. Upon comparing the displacements along the three main axes, it was evident that the displacement $U_{3}$ exhibited the most significant magnitude, with amplitudes 827 and 737 times larger than those of $U_{1}$ and $U_{2}$, respectively. The maximum displacement of $U_{3}$ measured 0.334 mm, exceeding the results obtained from the three-dimensional analysis shown in Figure 13b by 10.17%. This observation indicates a favorable level of accuracy in the recovery of the local displacement field.

It is noteworthy that the recovered local displacement within the unit cell was typically greater at the trough time compared to that at the peak time. This disparity can be attributed to the presence of the hierarchical honeycomb within the sandwich panel. The hierarchical honeycomb hindered the vibration of the sandwich panel at the trough time, resulting in an amplified amplitude. Conversely, at the peak time, the influence of the hierarchical honeycomb was relatively limited, leading to a smaller amplitude.

Table 4 shows the local stress field within the unit cell at the peak and trough times. The results clearly indicate that the local stress was mainly taken by the facesheets. Additionally, there existed a notable concentration of localized stress at the interface between the facesheet and the core layer, indicating high susceptibility to damage. The analysis outcomes for the local stress field hold paramount importance for fatigue failure analysis.

It is important to highlight that the local stress at the trough time of the forced vibration surpassed that at the peak time, a phenomenon ascribed to the internal structural attributes of the SP-HHH. The hierarchical honeycomb core within the panel restricted the propagation path of the vibration at the trough time, thereby impeding the transfer and dispersion of the vibrational energy. As a result, the vibrational energy accumulated within a confined region, leading to pronounced local stress at the trough time. Conversely, the influence of the hierarchical structure diminished during the peak time. At this juncture, the hierarchical honeycomb core exerted a lesser impact on vibration, enabling greater freedom for the dissemination and dispersion of vibrational energy. Consequently, the accumulation and concentration of local stress at the peak time were reduced.

## 4. Structural Parameter Analysis

This section investigates the effects of the length and thickness ratios of the first-order hierarchy on the dynamic characteristics of the SP-HHH under CCCC BCs using the 2D-EPM. A circular excitation area with a diameter of 20 mm located at the geometric center of the panel was subjected to a sinusoidal harmonic load of 1 MPa. The damping ratio was maintained at 0.01.

### 4.1. Microstructure-to-Hexagon Thickness Ratio

**Figure 14:**
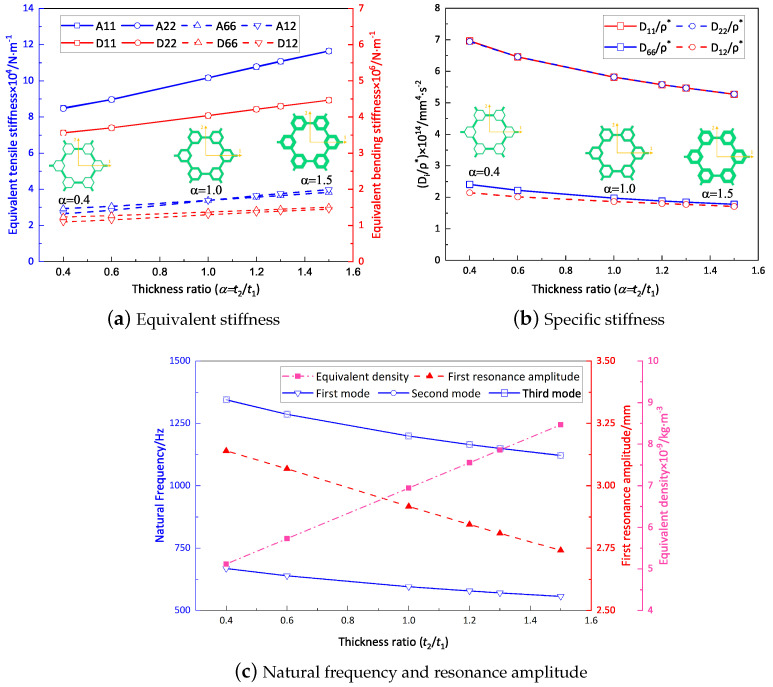
Effect of thickness ratio on the dynamic characteristics of the SP-HHH (the abscissa represents the microstructure-to-hexagon thickness ratio).

Figure 14a shows the variation curve for the equivalent stiffness with a thickness ratio of $t_{2}/t_{1}$ while the other parameters remain unchanged ($l_{1}=10\;\mathrm{mm},\;l_{2}=3\;\mathrm{mm}$). It is evident that the equivalent tensile and bending stiffness both increased gradually as the thickness ratio increased from 0.4 to 1.5. Figure 14b shows that the specific stiffness decreased with increasing thickness ratio. Furthermore, Figure 14c exhibits changes in natural frequencies and the first response amplitude with alterations in the thickness ratio. It is notable that the first three natural frequencies decreased gradually with increasing thickness ratio, accompanied by a gradual decrease in the first resonance amplitude. This decrease could be attributed to the increase in equivalent density, which was relatively greater than the resulting increase in equivalent stiffness due to the increase in the thickness ratio.

### 4.2. Microstructure-to-Hexagon Length Ratio

**Figure 15:**
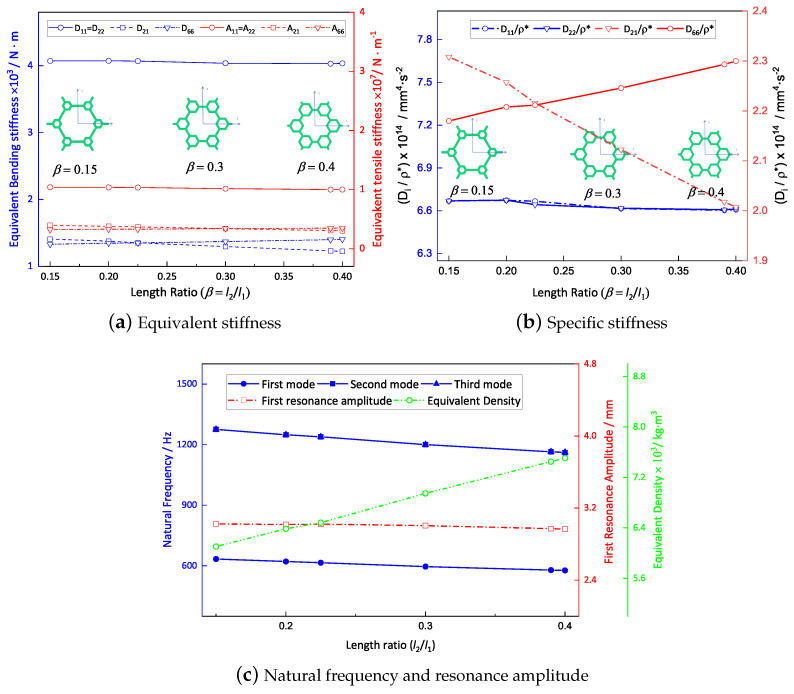
Effect of length ratio on dynamic characteristics of the SP-HHH (the abscissa represents the microstructure-to-hexagon length ratio).

Figure 15a presents the relationship between the microstructure-to-hexagon length ratio ($l_{2}/l_{1}$) and the equivalent stiffness in the 2D-EPM, while the other parameters were kept constant ($t_{1}=t_{2}=1\;\mathrm{mm}$). The findings demonstrate that the equivalent stiffness was uniform along both principal axes. This uniformity arose from a decrease in the region along the shorter side accompanied by a complete strengthening of the microstructure. Conversely, the region along the longer side increased but only experienced a partial reinforcement of the microstructure. These opposing effects counterbalanced each other, resulting in similar equivalent stiffness along the directions of both principal axes.

Figure 15b shows that there was no substantial change in the specific bending stiffnesses as the length ratio increased. However, the specific torsional stiffness, represented by $D_{66}/\rho^{*}$, exhibited a significant increase. This discrepancy can be primarily attributed to the longitudinal bending stiffness of the top and bottom facesheets of the hierarchical honeycomb sandwich panel, which predominantly influenced the specific bending stiffness. Consequently, alterations in the vertex microstructures in the core layer had minimal impact on the specific bending stiffness. Conversely, an augmentation in the length ratio resulted in an increased number of transverse connections within the core layer and improved their quality. This phenomenon subsequently enhanced the shear stiffness of the sandwich panel, thus significantly enhancing the overall torsional stiffness.

Figure 15c illustrates the variations in the first resonance amplitude and first three natural frequencies over the length ratio range of 0.15 to 0.40. The results indicate a decrease in all three natural frequencies as the length ratio increased, indirectly suggesting a continuous enhancement in the stiffness of the sandwich panel. Specifically, an increase in the specific stiffness led to a reduction in resonance amplitude.

### 4.3. Summary

To present the impact of the two ratios on the dynamic characteristics of the SP-HHH in a more intuitive manner, Table 5 displays the variations in the equivalent stiffness ($A_{11}$ and $D_{11}$), specific stiffness ($D_{11}/\rho^{*}$), fundamental frequency ($f_{1}$), and the first resonance amplitude ($R_{A}$) associated with these ratios. It is evident that the impact of the thickness ratio on the equivalent stiffness, frequency, and amplitude was considerably greater than that of the length ratio within a specific range. However, an increase in the thickness ratio could result in a more pronounced reduction in specific stiffness. In practical design, it is crucial to emphasize the thickness ratio that has a more pronounced influence on the dynamic characteristics of the SP-HHH. Furthermore, the findings suggest that the stiffness of the hierarchical honeycomb sandwich panel is mainly limited by the vertexes, and reinforcement of the vertexes can enhance the sandwich panel’s stiffness due to the even distribution of mass and materials across the entire sandwich panel.

## 5. Geometric Variation of the Vertex Cell

**Figure 16:**
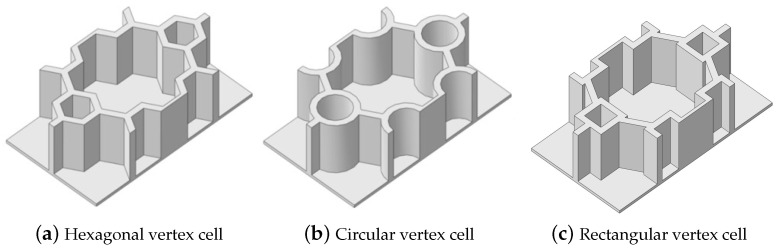
Hierarchical honeycomb with (**a**) hexagon, (**b**) circular, and (**c**) rectangular vertex cells.

The geometric variations of the vertex cells in hierarchical honeycomb structures play a crucial role in adjusting the overall performance. It is, therefore, imperative to acquire a comprehensive understanding of these variations to comprehend the behavior of the structure as a whole. This section investigates the impact of diverse vertex cell patterns on the dynamic characteristics of an SP-HHH. In addition to the mentioned self-similar hierarchical hexagonal honeycomb, two innovative hierarchical honeycombs representing circular and rectangular vertex cells were developed, as shown in Figure 16b,c. The length and thickness ratios of the three hierarchical honeycombs were all set to 0.3 and 1, respectively.

**Figure 17:**
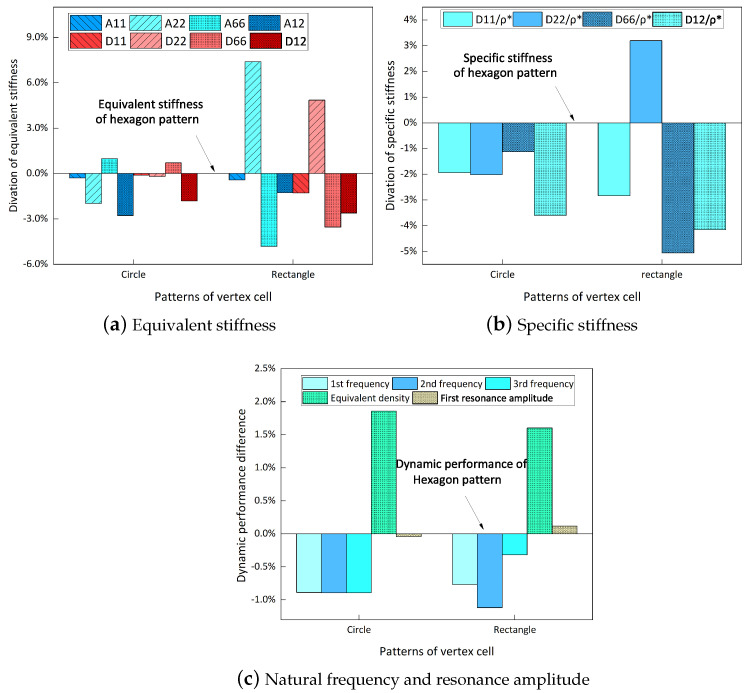
Effects of different vertex cell patterns on dynamic characteristics of SP-HHHs.

Figure 17 shows the effects of different vertex cell patterns on the dynamic characteristics of the SP-HHH. It reveals that the SP-HHH with hexagonal vertex cells displayed higher specific stiffness than the rectangular one (except $D_{22}/\rho^{★}$). Furthermore, both the specific stiffness and equivalent stiffness of the SP-HHH with circular vertex cells were inferior to those of the other two patterns. Figure 17c shows that the SP-HHHs with circular and rectangular vertex cells exhibited higher equivalent densities when compared to the one with hexagonal vertex cells, and the first three natural frequencies were smaller. However, the SP-HHH with circular vertex cells demonstrated a smaller first resonance amplitude than that the one with the hexagonal vertex cells. Hence, it can be concluded that the SP-HHH with hexagonal vertex cells has superior specific stiffness compared to those with circular and rectangular vertex cells, resulting in a more lightweight design and enhanced stiffness. Conversely, the SP-HHH with circular vertex cells exhibited a greater mass-to-stiffness ratio. As a result, it offers distinct advantages in terms of amplitude control.

Table 6 and Table 7 compare the local displacement and stress fields within the central unit cell of the sandwich panels with circle and rectangular vertex cells, respectively. Upon comparing the results presented in Table 3 and Table 4, it is evident that the local stresses had a most pronounced uneven distributed in both the core layer and facesheet of the unit cell with rectangular vertex cells. However, such an uneven stress distribution was significantly alleviated in the unit cell with hexagonal and circular vertex cells. Furthermore, a noticeable concentration of local stress occurred at the connection between the vertex unit and facesheet, with rectangular vertex cells displaying the most severe stress concentration. This phenomenon of stress concentration was substantially mitigated in hexagonal and circular vertex cells, resulting in enhanced bearing capacity and fatigue resistance. Consequently, hexagonal vertex cells offer a favorable balance between lightweight design and high strength, closely approximating the stress distribution state of circular vertex cells. This advantageous property contributes significantly to their superior stiffness and strength.

## 6. Conclusions

This study establishes an equivalent two-dimensional model for sandwich panels with a hierarchical hexagonal honeycomb by employing the variational asymptotic method. To verify the accuracy of the established model in terms of dynamic characteristics, free and forced vibration analyses of the SP-HHH were conducted. The obtained results indicate that the equivalent two-dimensional model can effectively and precisely analyze an SP-HHH’s dynamic characteristics. Through investigating the influence of the length and thickness ratios of the first-order hierarchy on the dynamic characteristics of the SP-HHH, the following conclusions were drawn:

(1) As the microstructure-to-hexagon thickness ratio increased, there was a concurrent increase in the panel’s equivalent stiffness. However, the first resonance amplitude and the first three natural frequencies exhibited a gradual decrease due to a correlated decrease in specific stiffness, as dictated by the thickness ratio;
(2) Increasing the microstructure-to-hexagon length ratio led to a decrease in the first resonance amplitude and the first three natural frequencies of the SP-HHH. However, the equivalent stiffness of the sandwich panel continued to increase. These findings suggest that the vertices of the hierarchical honeycomb structure are the weakest part, and strengthening them could help enhance the overall stiffness of the sandwich panel;
(3) The SP-HHH with hexagonal vertex cells demonstrated higher specific stiffness compared to configurations with circular and rectangular vertex cells, resulting in a more lightweight design and improved stiffness. In contrast, the SP-HHH with circular vertex cells exhibited a higher mass-to-stiffness ratio, providing distinct advantages in terms of amplitude control.

## Figures and Tables

**Table 1:** Comparison of the fist six natural frequencies predicted by the two models under different BCs. Unit: Hz.

Order	CCCF			CCFF			CFFF		
	3D-FEM	2D-EPM	Error	3D-FEM	2D-EPM	Error	3D-FEM	2D-EPM	Error
1	427.07	399.17	6.53%	396.05	415.60	4.94%	62.18	65.89	5.97%
2	705.24	694.17	1.57%	470.63	482.56	2.53%	151.67	150.92	0.49%
3	1110.60	1037.50	6.58%	767.35	752.38	1.95%	378.44	396.45	4.76%
4	1333.80	1342.50	0.65%	1075.30	1132.50	5.32%	481.62	460.35	4.42%
5	1401.10	1364.00	2.65%	1179.80	1224.00	3.75%	547.62	555.36	1.41%
6	2000.50	1972.20	1.41%	1388.90	1320.40	4.93%	950.17	932.42	1.87%

**Table 2:** Comparison of first six natural frequencies and vibration modes predicted by the two models under CCCC BCs.

Order	1	2	3	4	5	6
3D-FEM	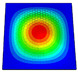	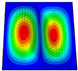	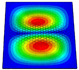	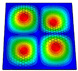	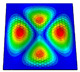	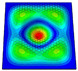
	635.79 Hz	1275.40 Hz	1277.30 Hz	1857.7 Hz	2239.40 Hz	2252.20 Hz
2D-EPM	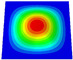	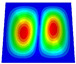	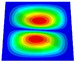	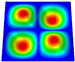	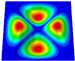	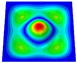
	595.83 Hz	1199.50 Hz	1199.60 Hz	1747.60 Hz	2111.20 Hz	2123.00 Hz
Error	6.3%	6.0%	6.1%	5.9%	5.7%	5.7%

**Table 3:** Recovered local displacement fields within the unit cell at the peak and trough times of the time-domain curve.

Displacement	Trough Time	Peak Time
*U*	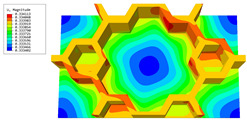	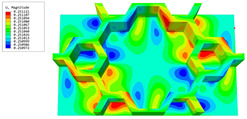
$U_{1}$	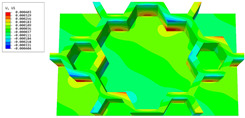	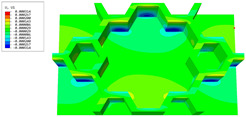
$U_{2}$	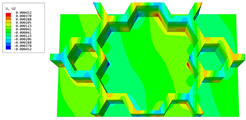	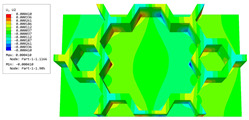
$U_{3}$	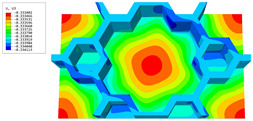	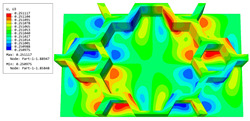

**Table 4:** Recovered local stress fields within the unit cell at the peak and trough times of the time-domain curve.

Stress	Trough Time	Peak Time
Von Mises	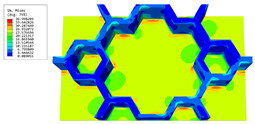	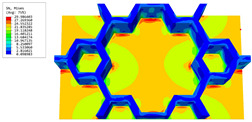
$\sigma_{11}$	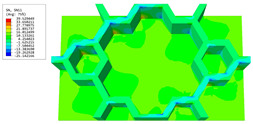	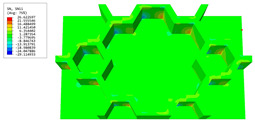
$\sigma_{22}$	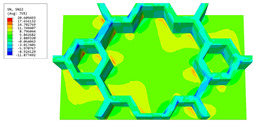	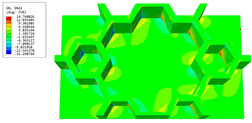
$\sigma_{33}$	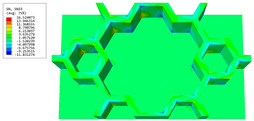	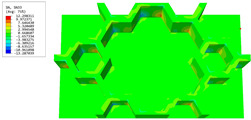

**Table 5:** Influence of geometric parameters on the dynamic characteristics of the SP-HHH.

Ratios	Range	$\Delta A_{11}$	$\Delta D_{11}$	$\Delta D_{11}/\rho^{*}$	$\Delta \rho^{*}$	$\Delta f_{1}$	$\Delta R_{A}$
Thickness ratio	0.4–1.5	27.0%	30.8%	−24.3%	65.4%	−16.7%	−12.7%
Length ratio	0.15–0.4	6.1%	6.4%	6.4%	33.7%	−7.0%	−1.9%

**Table 6:** Recovered local displacement fields within the central unit cell of sandwich panels with circular and rectangular vertex cells at the trough time of the time-domain curve.

Displacement	Circular Vertex Cell	Rectangular Vertex Cell
*U*	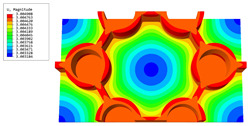	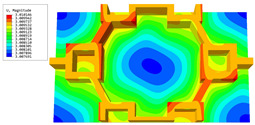
$U_{1}$	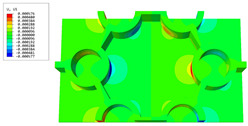	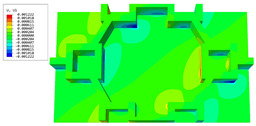
$U_{2}$	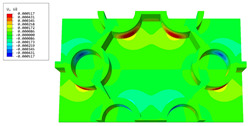	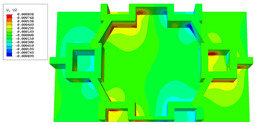
$U_{3}$	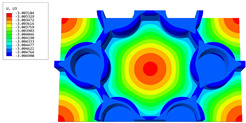	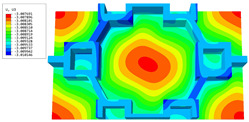

**Table 7:** Recovered local stress fields within the central unit cell of sandwich panels with circular and rectangular vertex cells at the trough time of the time-domain curve.

Stress	Circular Vertex Cell	Rectangular Vertex Cell
$\sigma_{\mathrm{Mises}}$	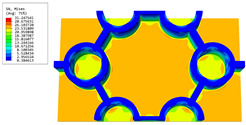	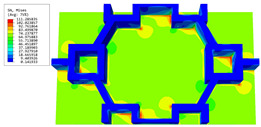
$\sigma_{11}$	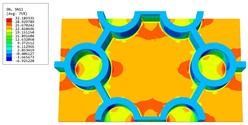	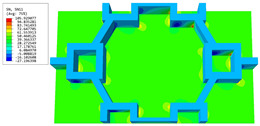
$\sigma_{22}$	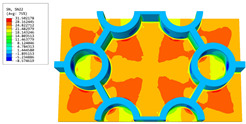	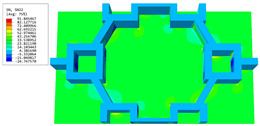
$\sigma_{33}$	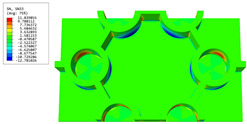	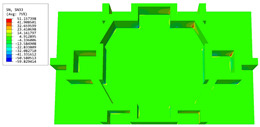

## Data Availability

Data available upon request due to restrictions; e.g., privacy or ethical. The data presented in this study are available upon request from the corresponding author. The data are not publicly available due to subsequent analyses and publications.

## Abbreviations

The following symbols were used in this paper:

$x_{i},y_{i}$	Macro- and micro-coordinates, respectively
ζ	Micro–macro scale ratio
$u_{i},{\bar{u}}_{i}$	Displacements of the 3D-FEM and 2D-EPM, respectively
〈·〉	Integral over the volume domain of the unit cell
$\chi_{i}$	Warping function
$E_{e},E_{s},E_{t}$	Strain components of the 3D-FEM
$l_{1},l_{2}$	Side lengths of the microstructure and hexagon, respectively
$t_{1},t_{2}$	Wall thicknesses of the microstructure and hexagon, respectively
$t_{f},t_{c}$	Thicknesses of the facesheet and core layer, respectively
*s*	Reference surface of the sandwich panel
$\frac{u_{\Omega }}{\Omega }$	Strain energy density
$\tau_{i},\beta_{i},f_{i}$	Traction forces on the top and bottom surfaces of the plate and body force
*V*	Absolute velocity measured on the deformed reference surface
ω	Inertial angular velocity
$\rho ,\rho^{*}$	Density and equivalent density of the 3D-FEM and 2D-EPM, respectively
$\rho ,\rho^{*}$	Density and equivalent density of the 3D-FEM and 2D-EPM, respectively
$\lambda_{i}$	Lagrange multipliers
$\delta {\bar{W}}_{2D},\delta {\bar{W}}^{*}$	Virtual work independent of and related to the warping function
